# A gene sets approach for identifying prognostic gene signatures for outcome prediction

**DOI:** 10.1186/1471-2164-9-177

**Published:** 2008-04-16

**Authors:** Seon-Young Kim, Yong Sung Kim

**Affiliations:** 1 Human Genomics Laboratory, Functional Genomics Research Center, KRIBB, Daejeon 305-806, Korea

## Abstract

**Background:**

Gene expression profiling is a promising approach to better estimate patient prognosis; however, there are still unresolved problems, including little overlap among similarly developed gene sets and poor performance of a developed gene set in other datasets.

**Results:**

We applied a gene sets approach to develop a prognostic gene set from multiple gene expression datasets. By analyzing 12 independent breast cancer gene expression datasets comprising 1,756 tissues with 2,411 pre-defined gene sets including gene ontology categories and pathways, we found many gene sets that were prognostic in most of the analyzed datasets. Those prognostic gene sets were related to biological processes such as cell cycle and proliferation and had additional prognostic values over conventional clinical parameters such as tumor grade, lymph node status, estrogen receptor (ER) status, and tumor size. We then estimated the prediction accuracy of each gene set by performing external validation using six large datasets and identified a gene set with an average prediction accuracy of 67.55%.

**Conclusion:**

A gene sets approach is an effective method to develop prognostic gene sets to predict patient outcome and to understand the underlying biology of the developed gene set. Using the gene sets approach we identified many prognostic gene sets in breast cancer.

## Background

Many researchers have studied the feasibility of gene expression profiling to improve the prognosis of cancer patients and have shown that gene expression signatures can better predict the outcome of cancer patients than conventional clinical criteria in many cancer types [1-4]. A few of the discovered signatures are now in large clinical trials to confirm their prognostic value [5,6]. However, there are also concerns about the usefulness of the gene expression signatures because several problems remain unresolved [7-9]. These problems include poor overlap among discovered gene signatures, the unstable nature of gene expression signatures, and poor performance of signatures when applied to other datasets [7,9-11].

Researchers have applied either top-down or bottom-up approaches to discover prognostic gene signatures [12]. Most researchers have used the top-down approach in which samples are split into training and testing sets and gene signatures are developed by discovering genes that show a high correlation between expression and clinical information [2,13-19]. In the bottom-up approach, gene signatures developed from other biological models are applied to gene expression datasets to classify patients into clinically distinct groups [12,20]. One advantage of the bottom-up approach is that it affords a straightforward understanding of the underlying biological process behind the discovered gene signature [12]. Similarly, the recently developed gene set enrichment analysis (GSEA) and similar methods are promising tools for high-throughput data analysis. These methods enable researchers to identify significantly changed biological themes and pathways from gene expression data by observing changes in expression using pre-defined gene sets [21,22]. Another method, named globaltest, was recently developed to test the association of a pathway with survival using gene expression data [23].

A gene signature is useless if it works well only on the dataset from which it was developed. Thus, recent work includes external validation of developed signatures as a necessary step that will reinforce the applicability of gene signatures to other datasets [14,15,24]. Here, we suggest a simple but very effective approach to identify gene signatures that are prognostic in multiple datasets. Rather than developing a signature from one dataset and validating it in other datasets, we suggest simultaneously testing multiple pre-defined gene signatures on multiple datasets to identify signatures that are prognostic in as many independent datasets as possible. By exhaustively testing all combinations of gene sets and datasets, our approach guarantees that the best gene signature will be identified among a pool of pre-defined gene sets. Moreover, our approach will enable better understanding of the underlying biology of disease by observing the patterns of association between gene expression and clinical parameters at multiple gene set levels.

In this work, we applied a bottom-up, gene sets approach to multiple datasets to determine gene signatures for prognosis of breast cancer patients. We chose breast cancer because there are several high-quality breast cancer gene expression datasets with survival or recurrence information. Our goal was to identify prognostic gene signatures useful in as many independent datasets as possible. For this, we collected 12 different datasets comprising 1,756 tumor samples and prepared 2,411 gene sets from diverse sources including gene ontology, biological pathways, and previously identified prognostic gene signatures for breast cancer. For each gene set, we performed survival analysis to test if the gene set could classify patients into clinically distinct groups. We also evaluated each gene set for the accuracy of outcome prediction.

## Results

### Selection of gene sets for prognosis of survival or recurrence

**Figure 1:**
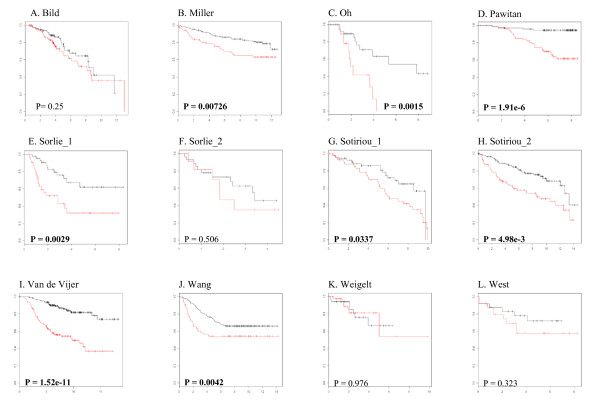
**Kaplan-Meier survival curves for the two prognostic classes of breast cancers.** In each dataset, patients were divided into two groups (poor and good prognostic groups) based on the gene expression pattern in the 11823860_ST2 gene set, and their survival or recurrence proportions were then plotted. The log-rank test was used to infer the statistical significance of survival or recurrence differences between the two groups. In each graph, the x-axis represents overall or relapse-free survival years and the y-axis represents the proportion of overall survival (A, B, C, D, E, F, I, and K) or relapse-free survival (G, H, J, and L). Black indicates poor prognosis and red indicates good prognosis.

Analysis of 12 datasets (Table 1) with 2,411 gene sets (Table 2) including 32 gene sets previously identified as prognostic in breast and other cancers (Table 3) revealed that many of the gene sets related to cell cycle or proliferation were best discriminating between good and poor prognosis groups. Table 4 presents the 20 most highly prognostic gene sets identified by two-means clustering of samples. Most of these top gene sets were related to cell cycle, mitosis, proliferation, and DNA replication as well as gene sets previously identified as prognostic in breast cancer such as 11823860_ST2, 17076897_ADF3, and 16478745_ST1 (Table 4). Kaplan-Meier plots of 12 datasets showed that the 11823860_ST2 gene set classified patients into two groups (poor or good prognosis) according to differences in survival or recurrence in eight of 12 datasets (Figure 1). Because breast cancers are heterogeneous and may comprise three to six subtypes [25-27], we also applied k-means clustering with k = 3, 4, 5, and 6 to each dataset to divide samples into three, four, five, and six subtypes respectively and performed log-rank test to infer the significance of differences in survival between the groups. Again, we found that gene sets related to cell cycle or proliferation were best discriminating between groups with different clinical outcomes (Additional data file 1, Supplementary Table 1, 2, 3, and 4). The 11823860_ST2 gene set, which was ranked as the first in two-means clustering analysis (Table 4), was ranked as the first in four (Supplementary Table 2) and the fifth in three (Supplementary Table 1) and the tenth in five and six-means clustering (Supplementary Table 3 and 4).

**Table 1:** Breast cancer datasets analyzed in this study

Study	Platform	Samples	Data source
Bild	Affymetrix	169	*GSE3143
Miller	Affymetrix	251	GSE3494
Oh	Oligos Agilent	67	
Pawitan	Affymetrix	159	GSE1456
Sorlie_1	Spotted cDNA	76	GSE3193
Sorlie_2	Spotted cDNA	39	
Sotiriou_1	Spotted cDNA	99	
Sotiriou_2	Affymetrix	187	GSE2990
Van de Vijver	oligos Agilent	295	
Wang	Affymetrix	286	GSE2034
Weigelt	Oligos Agilent	79	
West	Affymetrix	49	

Total		1756	

*GSE: gene expression series number in GEO (gene expression omnibus)

**Table 2:** Number of gene sets in each category

Category	Number
GO Biological Process (BP)	735
GO Molecular Functions (MF)	648
Biological Pathways	198
InterPro Domains	798
Breast and other Cancer Signatures	32

Total	2411

**Table 3:** Thirty-two prognostic gene sets prepared from published reports

Gene set	Number (reported)	Number (unique)	Reference
*11823860_ST2	231	164	van't Veer et al. [13]
11823860_ST3	2,460	1,818	van't Veer et al. [13]
11823860_ST4	430	314	van't Veer et al. [13]
12490681_70	70	50	van de Vijver [1]
12747878_ST2	177	144	Huang et al. [52]
12747878_ST3	168	160	Huang et al. [52]
12917485_ST6	606	564	Sotiriou et al. [18]
12917485_ST7	137	126	Sotiriou et al. [18]
12917485_ST8	706	635	Sotiriou et al. [18]
12917485_ST9	485	402	Sotiriou et al. [18]
14737219_CSR	512	459	Chang et al. [3]
14737219_USR	677	611	Chang et al. [3]
15034139_T2	45	31	Zhao et al. [53]
15073102_4	4	4	Glinsky et al. [54]
15073102_6	6	6	Glinsky et al. [54]
15073102_13	12	12	Glinsky et al. [54]
15073102_14	14	14	Glinsky et al. [54]
15591335_F1	21	21	Paik et al. [6]
15721473_T3	76	68	Wang et al. [2]
15931389_T3_stem	11	11	Glinsky et al. [55]
15931389_ST2_14	14	14	Glinsky et al. [55]
15931389_ST2_CNS	11	11	Glinsky et al. [55]
16141321_SDC2	500	398	Miller et al. [19]
16273092_catenin	98	76	Bild et al. [20]
16273092_E2F3	298	238	Bild et al. [20]
16273092_myc	332	192	Bild et al. [20]
16273092_RAS	348	248	Bild et al. [20]
16273092_SRC	75	58	Bild et al. [20]
16280042_AF1	64	61	Pawitan et al. [16]
16478745_ST1	242	207	Sotiriou et al. [15]
16707453_ST3	101	86	Schuetz et al. [56]
17076897_ADF3	52	52	Teschendorff et al. [24]

*Eight-digit number represents PubMed id of a reference

**Table 4:** Top 20 prognostic gene sets identified by two-means clustering in breast cancer gene expression datasets

Gene set	*category	Bild	Miller	Oh	Pawitan	Sorlie_1	Sorlie_2	Sotiriou_1	Sotiriou_2	van de Vijver	Wang	Weigelt	West	^#^freq	^%^mean
11823860_ST2	BR	1.32	7.21	10.02	22.68	8.87	0.44	4.51	8.18	45.51	8.19	0	0.97	8	9.83
mitotic checkpoint	BP	7.51	13.34	2.91	13.57	0.07	0.03	4.08	9.59	30.78	12.49	0.01	3.57	7	8.16
Cell_cycle_KEGG_GenMAPP	PW	7.2	12.05	2.08	11.46	4.28	0.31	2.75	9.33	40.26	6.93	0.01	0.03	7	8.06
cell division	BP	4.37	10.47	3.46	13.81	6.05	0	2.14	7.69	32.14	15.18	0.02	0.06	7	7.95
cation efflux protein	IP	7.94	9.69	2.16	15.77	4.16	2.41	1.96	10.45	24.69	10.04	0.51	0.21	7	7.5
cyclin, C-terminal	IP	3.88	15.25	6.72	16.84	5.65	0.07	2.64	3.84	21.12	7.7	0.69	0.23	7	7.05
DNA repair	BP	2.04	7.15	4.58	9.13	0.09	0.02	6.61	8.4	35.15	6.97	0.13	1.53	7	6.82
cyclin, N-terminal domain	IP	3.4	15.66	2.50	10.93	5.5	0.3	4.37	3.91	26.72	7.28	1.03	0.03	7	6.8
protein tyrosine phosphatase activity	MF	9.45	4.03	8.29	9.19	4.51	0.55	2.55	0.46	24.1	9.25	0	2.84	7	6.27
protein domain specific binding	MF	6.56	5.32	0	10.73	0.14	1.24	12.14	6.81	15.53	2.69	10.09	0.66	7	5.99
DNA metabolism	BP	4.08	8.01	1.81	10.15	0.06	0.17	5.06	4.88	26.2	8.88	0.64	0.3	7	5.85
identical protein binding	MF	0.18	8.04	8.35	9.55	0.12	5.3	8.79	4.09	19.64	0.01	0.97	0.12	7	5.43
water transport	BP	0.01	10.23	5.85	5.26	0.45	5.25	1.57	0.42	3.97	0.91	6.13	5.14	7	3.77
17076897_ADF3	BR	3.15	14.49	4.06	19.84	0.1	2.59	2.31	12.03	48.93	18.04	0	2.39	6	10.66
mitosis	BP	6.45	13.81	2.22	16.95	1.35	0.1	2.23	9.05	37.87	11.43	0	0.16	6	8.47
16478745_ST1	BR	5.49	10.52	3.2	13.32	1.04	0	2.55	11.64	39.05	12.51	0.29	0.53	6	8.35
Pyrimidine metabolism_KEGG	PW	4.28	7.84	4.35	25.6	0.61	0.46	2.07	8.04	42.75	3.12	0	0.77	6	8.32
14737219_USR	BR	4.27	10.25	3.72	13.61	0.99	0.06	1.96	10.86	39.37	11.4	0.16	0.2	6	8.07
cytokinesis	BP	2.6	8.9	3.91	17.06	0.03	0.91	0.22	8.68	48.68	5.24	0.16	0.17	6	8.05
14737219_CSR	BR	0.51	10.7	3.13	15.5	7.45	0.08	4.25	1.43	39.65	7.32	0.38	2.3	6	7.73

Values are chi-square values from log-rank test.

#frequency: The number of cases in which chi-square value is over 3.84

*category: BP-GO Biological Processes, BR-Breast cancer prognostic signatures, MF-GO Molecular Function, PW-KEGG and GenMAPP pathways, IP-InterPro domains

^%^mean: Mean of 12 chi-square values

### Unadjusted and adjusted hazard ratios

We then calculated unadjusted hazard ratios for three selected gene sets within the 12 datasets (Table 5). These three gene sets showed significant (P < 0.05) unadjusted hazard ratios in six or seven of the 12 datasets irrespective of microarray platforms. For example, the Sotiriou_2, Wang, and Pawitan datasets used the Affymetrix U133A platform, the van de Vijver dataset used Agilent oligomers, and the Sorlie_1 dataset used cDNA arrays. This confirms that many gene sets related to cell cycle and proliferation are prognostic irrespective of the microarray platform. We also calculated adjusted hazard ratios for the 11823860_ST2 gene set in the three datasets (Sotiriou_2, van de Vijver, and Sorlie_1) for available clinical parameters such as grade, lymph node status, tumor size, age, and estrogen receptor (ER) status (Additional data file 2, Supplementary Table 5, 6 and 7). The 11823860_ST2 gene set proved significant even after adjustment for other clinical parameters in the three datasets, verifying that the 11823860_ST2 gene set contains additional prognostic value over existing prognostic clinical parameters.

**Table 5:** Hazard ratios and P values for the top three gene signatures in 12 datasets

Datasets	11823860_ST2	Mitotic checkpoint	Cell_cycle_KEGG
Bild	6.35 *^#^**(1.23–32.2) p = 0.0256**	2.88 (0.686–12.1) p = 0.148	1.13 (0.407–3.11) p = 0.819
Miller	1.29 (0.297–5.63) p = 0.731	0.942 (0.269–3.3) p = 0.925	1.37 (0.547–3.41) p = 0.504
Oh	4.72 (0.834–26.7) p = 0.0794	3.87 (0.792–18.9) p = 0.0944	2.07 (0.728–5.9) p = 0.172
Pawitan	**34.6 (4.94–242) p = 3.57e-4**	**11.9 (2.84–49.9) p = 7.1e-4**	**5.21 (1.97–13.8) p = 8.6e-4**
Sorlie_1	**6.84 (1.75–26.7) p = 0.00568**	**4.73 (1.46–15.3) p = 0.00953**	**2.07 (1.07–4.01) p = 0.0312**
Sorlie_2	3.28 (0.29–46.9) p = 0.381	1.99 (0.308–12.8) p = 0.471	1.33 (0.319–5.57) p = 0.695
Sotiriou_1	27.3 (2.60–287) p = 0.0582	**64.2 (2.22–1854) p = 0.0153**	**5.58 (1.19–26.20 p = 0.0296**
Sotiriou_2	**5.22 (1.63–16.8) p = 0.00549**	**3.13 (1.17–8.42) p = 0.0234**	**2.6 (1.24–5.44) p = 0.0113**
Van de Vijver	**62.3 (17.7–219) p = 1.12e-10**	**8.8 (4.18–18.5) p = 1.05e-8**	**4.03 (2.37–6.85) p = 2.73e-7**
Wang	**7.48 (2.78–20.1) p = 6.92e-5**	**2.73 (1.22–6.1) p = 0.0144**	**3.78 (1.89–7.55) p = 1.66e-4**
Wiegelt	2.00 (0.152–26.0) p = 0.597	1.40 (0.15–13.0) p = 0.769	1.25 (0.19–3.38) p = 0.764
West	15.5 (0.73–329) p = 0.788	5.56 (0.635–12.1) p = 0.121	**4.27 (1.20–15.1) p = 0.0246**

*Values in parenthesis are 95% confidence intervals

^#^Bolded data entries are significant at P < 0.05.

### Accuracy of outcome prediction

We then analyzed the accuracy of patient outcome prediction for each of the 2,411 gene sets. Initially, we tested five algorithms – nearest centroid, diagonal linear discriminant analysis (DLDA), compound covariate predictor, one-nearest and three-nearest neighbor predictor [28] and found that in our datasets nearest centroid and DLDA methods performed better than the others (data not shown) with similar performance to each other. For convenience, we used the nearest centroid method in subsequent analysis. With six large datasets containing more than 100 samples, we estimated the prediction accuracy of each gene set by external validation. We measured prediction accuracy for each pair of 30 training-testing datasets and for a total of 30 predictions (Table 6). The best gene set was the gene set 11823860_ST2, with prediction accuracy, sensitivity, and specificity of 67.55%, 70.56%, and 57.16%, respectively (Tables 6 and 7). The individual prediction accuracy with the 11823860_ST2 gene set was as high as 0.7464 when the training-testing pair was Pawitan-van de Vijver and as low as 0.54 74 when the training-testing pair was Wang-Bild (Table 6). The individual prediction accuracy was not related to the differences in microarray platforms or patient characteristics (data not shown). We also analyzed the accuracy of patient outcome prediction with nine datasets with more than ten samples for each of the two groups. Again, the gene set 11823860_ST2 was the best with a prediction accuracy, sensitivity, and specificity of 0.6578, 0.6895, and 0.566, respectively (Additional data file 3, Supplementary Table 8).

**Table 6:** Prediction accuracy of the 11823860_ST2 gene set in external validation

training	testing	*GTG	GTP	PTG	PTP	**accuracy	sensitivity	specificity
Bild	Miller	128	49	17	19	0.6901	0.7232	0.5278
Bild	Pawitan	89	41	7	15	0.6842	0.6846	0.6818
Bild	Sotiriou_2	85	32	11	17	0.7034	0.7265	0.6071
Bild	Van de Vijver	165	67	11	37	0.7214	0.7112	0.7708
Bild	Wang	128	55	42	51	0.6486	0.6995	0.5484
Miller	Bild	37	24	17	17	0.5684	0.6066	0.5
Miller	Pawitan	84	46	6	16	0.6579	0.6462	0.7273
Miller	Sotiriou_2	77	40	7	21	0.6759	0.6581	0.75
Miller	Van de Vijver	165	67	11	37	0.7214	0.7112	0.7708
Miller	Wang	125	58	42	51	0.6377	0.6831	0.5484
Pawitan	Bild	43	18	19	15	0.6105	0.7049	0.4412
Pawitan	Miller	133	44	19	17	0.7042	0.7514	0.4722
Pawitan	Sotiriou_2	87	30	11	17	0.7172	0.7436	0.6071
Pawitan	Van de Vijver	173	59	12	36	0.7464	0.7457	0.75
Pawitan	Wang	135	48	51	42	0.6413	0.7377	0.4516
Sotiriou_2	Bild	38	23	18	16	0.5684	0.623	0.4706
Sotiriou_2	Miller	129	48	19	17	0.6854	0.7288	0.4722
Sotiriou_2	Pawitan	86	44	10	12	0.6447	0.6615	0.5455
Sotiriou_2	Van de Vijver	164	68	12	36	0.7143	0.7069	0.75
Sotiriou_2	Wang	131	52	43	50	0.6558	0.7158	0.5376
Van de Vijver	Bild	41	20	21	13	0.5684	0.6721	0.3824
Van de Vijver	Miller	136	41	21	15	0.7089	0.7684	0.4167
Van de Vijver	Pawitan	99	31	12	10	0.7171	0.7615	0.4545
Van de Vijver	Sotiriou_2	88	29	15	13	0.6966	0.7521	0.4643
Van de Vijver	Wang	141	42	54	39	0.6522	0.7705	0.4194
Wang	Bild	34	27	16	18	0.5474	0.5574	0.5294
Wang	Miller	123	54	14	22	0.6808	0.6949	0.6111
Wang	Pawitan	81	49	6	16	0.6382	0.6231	0.7273
Wang	Sotiriou_2	76	41	7	21	0.669	0.6496	0.75
Wang	Van de Vijver	154	78	8	40	0.6929	0.6638	0.8333

Total		3175	1325	559	746	0.6755	0.7056	0.5716

*GTG – Good prognosis group predicted as Good; GTP – Good prognosis group predicted as Poor; PTG – Poor prognosis group predicted as Good; PTP – Poor prognosis group predicted as poor

**accuracy = (GTG+PTP)/(GTG+GTP+PTG+PTP); sensitivity = GTG/(GTG+GTP); specificity = PTP/(PTG+PTP)

**Table 7:** Top 20 gene sets with high prediction accuracy (analysis with six datasets)

Gene set	category	GTG	GTP	PTG	PTP	accurary	sensitivity	specificity
11823860_ST2	br	3175	1325	559	746	0.6755	0.7056	0.5716
transferase activity	mf	3264	1236	658	647	0.6737	0.7253	0.4958
ligase activity	mf	3204	1296	633	672	0.6677	0.712	0.5149
11823860_ST3	br	3200	1300	632	673	0.6672	0.7111	0.5157
transcription factor activity	mf	3268	1232	701	604	0.667	0.7262	0.4628
16141321_SDC2	br	3169	1331	607	698	0.6661	0.7042	0.5349
oxidoreductase activity	mf	3209	1291	648	657	0.666	0.7131	0.5034
14737219_CSR	br	3165	1335	606	699	0.6656	0.7033	0.5356
12917485_ST9	br	3162	1338	611	694	0.6643	0.7027	0.5318
catalytic activity	mf	3209	1291	661	644	0.6637	0.7131	0.4935
RNA polymerase II transcription factor activity	mf	3235	1265	689	616	0.6634	0.7189	0.472
transport	bp	3186	1314	645	660	0.6625	0.708	0.5057
transcription	bp	3241	1259	701	604	0.6624	0.7202	0.4628
transporter activity	mf	3171	1329	631	674	0.6624	0.7047	0.5165
14737219_USR	br	3094	1406	555	750	0.6622	0.6876	0.5747
12917485_ST7	br	3140	1360	602	703	0.662	0.6978	0.5387
ATP binding	mf	3185	1315	647	658	0.662	0.7078	0.5042
kinase activity	mf	3205	1295	669	636	0.6617	0.7122	0.4874
metabolism	bp	3199	1301	666	639	0.6612	0.7109	0.4897
regulation of progression through cell cycle	bp	3108	1392	575	730	0.6612	0.6907	0.5594

*category: br – breast and other cancer gene set; mf – molecular functions; bp – biological processes

**GTG – Good prognosis group predicted as Good; GTP – Good prognosis group predicted as Poor; PTG – Poor prognosis group identified as Good; PTP – Poor prognosis group identified as Poor

^ accuracy = (GTG+ PTP)/(GTP+GTP+PTG+PTP); sensitivity = GTG/(GTG+GTP); specificity = PTP/(PTG+PTP)

### Best gene sets for prediction accuracy differ from those for prognosis

**Figure 2:**
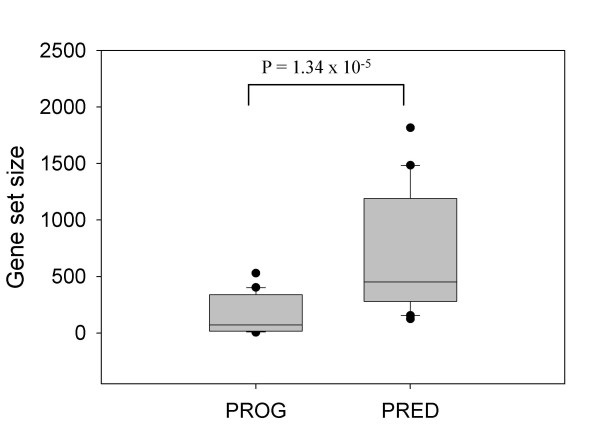
**Comparison of gene set sizes between best prognostic gene sets (group 1) and best gene predictive sets (group 2).** The number of genes in top 20 gene sets for group discrimination (PROG) and top 20 gene sets for prediction accuracy (PRED) is box plotted. P-value was inferred from an unpaired *t*-test.

Comparison of the top 20 prognostic gene sets for breast cancer survival (Table 4) with the top 20 gene sets with high prediction accuracy (Table 7) showed only three common gene sets (11823860_ST2, 14737219_USR, and 14737219_CSR). Interestingly, the gene sets shown in Table 7 were, in general, from higher categories in the gene ontology hierarchy, including transferase activity (MF), transcription factor activity (MF), transport (BP), and transcription (BP). Because gene sets in higher categories have more genes than those in lower categories, we reasoned that there might be a significant difference in gene set size between the gene sets in Table 4 and Table 7. Thus, we compared the distribution of gene set sizes between the top 20 prognostic gene sets for survival (designated as prognosis gene sets, Table 4) and the top 20 gene sets with high prediction accuracy (designated as predictor gene sets, Table 7) and found a significant difference in sizes between prognosis and predictor gene sets (Figure 2; P = 1.34 × 10^-5 ^by unpaired *t*-test). The sizes of the top 20 prognosis gene sets ranged from 6 to 530 with a mean of 155.5 and a median of 72.5, whereas the sizes of the top 20 predictor gene sets ranged from 125 to 1,817 with a mean of 674.15 and a median of 502.5 (Figure 2). The trend was repeatedly observed when we varied the number of top n prognosis and predictor gene sets (n = 10, 50, 100, 150, and 200) for comparison. The P-values by unpaired *t*-test to compare the difference in sizes between the two gene sets were 2.42 × 10^-3 ^(n = 10), 6.46 × 10^-8 ^(n = 50), 3.34 × 10^-7 ^(n = 100), 3.02 × 10^-8 ^(n = 150), and 4.55 × 10^-8 ^(n = 200), respectively

## Discussion

We have shown that a gene sets approach is effective in identifying prognostic gene sets over multiple gene expression datasets. We identified 11823860_ST2 gene set as the best prognostic gene set for breast cancer patients.

Our gene sets approach is fundamentally different from previous methods in that our method doesn't try to build a single gene set from gene expression and clinical data as previous methods did [2,3,13]. Instead, our method begins from multiple gene sets and datasets and exhaustively searches for the best gene set among the given gene sets. As more gene sets and datasets accumulate, our method always finds out a better gene set than before. Another advantage of our gene sets approach is that it assists us to understand the underlying biology of the clinical outcome because many gene sets are prepared using biological knowledge such as pathways, gene ontology, and protein domains [12,21,22]. In the analysis of breast cancer datasets, cell cycle or proliferation gene sets were the best for prognosis of survival or recurrence as judged by the log-rank test (Table 4). This result is in agreement with many previous studies showing that cell proliferation signatures are the best predictors of prognosis of breast cancer patients [1,2,12-16,18,24,29,30].

Because poor overlap among independently developed prognostic gene sets has raised concerns over this type of diagnostic tool [10,11], we examined the degree of overlap among the top 20 prognostic gene sets identified in our study. Again, we found relatively poor overlap among them, thus confirming previous results (data not shown). However, poor overlap among gene sets may not be as serious a problem as previously thought if different gene sets represent similar biological pathways and are congruent on outcome prediction [30-32]. This point was recently emphasized by Fan et al. [26] who showed congruence among four different gene expression-based predictors for breast cancer.

Pepe et al. [33] emphasized that strong statistical associations between prognostic markers and clinical outcomes do not necessarily imply good discriminative power of the marker. Thus, instead of reporting odds ratios or hazards ratios, one should report an objective prediction accuracy to prove the usefulness of the marker as a diagnostic, prognostic, or screening tool [33-35]. As such, we calculated the prediction accuracy of each gene set using six datasets containing over 100 samples. We emphasize that we performed only external validation to avoid over-fitted estimation of prediction accuracy. While Michiels et al. [7] showed that five of the seven datasets they analyzed did not classify patients better than by chance, at least for breast cancer, all six datasets that we analyzed classified patients even though we only used external validation.

When we prepared 2,411 gene sets, we included 32 gene sets previously identified as prognostic in breast and other cancers to evaluate their performance in multiple gene expression datasets. Among the included gene sets are the 70-gene signature (12490681_70 in Table 3) [1,13], 76-gene signature (15721473_T3 in Table 3) [2], 21-gene signature (15591335_F1 in Table 3) [6], and wound healing signature (14737219_CSR in Table 3) [3,12] (Table 3). Through various analyses, we identified the 11823860_ST2 gene set as the best prognostic gene set in breast cancer. The 11823860_ST gene set was the best in two and four-means clustering and also in outcome prediction (Table 4, 6, Supplementary Table 2 in Additional data file 1, and Supplementary Table 8 in Additional data file 3). The 11823860_ST2 gene set was also ranked high in three, five, and six-means clustering (Supplementary Table 1, 3, and 4 in Additional data file 1). The 11823860_ST2 gene set was originally identified as 231 genes significantly associated with clinical outcomes of 78 node-negative, untreated, and young patients with an age at diagnosis less than 55 years in a supervised analysis [13]. But, in our analysis with 12 datasets, the 11823860_ST2 gene set was also prognostic in independent patients with diverse clinical characteristics (both node-negative and positive, both treated and untreated patients of all ages), which was previously confirmed [1,18]. Also, the 11823860_ST2 gene set was prognostic in most datasets irrespective of the used microarray platforms.

In van't Veer et al. [13]'s work, 11823860_ST2 gene set was reduced to the famous 70-gene signature by optimizing the number of genes for maximum accuracy in leave-one-out cross validation [13]. The 70-gene signature has been validated in subsequent works and now undergoes a large scale prospective clinical trial [1]. But, our results indicate that using 231 genes might be better than using the 70-gene signature. Then, why 11823860_ST2 gene set performed better than the 70-gene signature? One reason is because we included in our analysis 12 different datasets produced using diverse microarray platforms with different gene contents. In this situation, gene sets containing many genes are likely to perform better than gene sets with a small number of genes because a greater proportion of prognostic genes are consistently present across all platforms. Indeed, the 11823860_ST2 gene set contains many genes (for example, cyclin E2, MCM6, MMP9, MP1, RAB6B, PK429, ESM1, and FLT1), in addition to 70 genes, involved in processes such as cell cycle, invasion and metastasis, angiogenesis, and signal transduction, processes up-regulated in poor prognosis group [13]. The tendency of gene sets with high prediction accuracy (Table 7) having more genes than prognostic gene sets identified by log-rank test (Table 4) may be explained in the same way (Figure 2).

One concern in our strategy is that by taking a certain number of pre-defined gene sets, it may just happen that one gene set will turn out significant. However, because the two procedures we perform, log-rank test and the estimation of prediction accuracy, evaluate at individual gene-set level whether a gene set is prognostic or not, we suppose that our method can effectively handle false positive predictions. Thus, even if a gene set is identified as the best among pre-defined gene sets, the two procedures, log-rank test and prediction accuracy, will evaluate if the identified gene set is significant or not.

Many microarray-based molecular studies have been criticized as noisy discovery due to problems such as small sample size, inappropriate statistical analysis leading to over-fitting of data, lack of independent validation, or validation with too small set [9,36,37]. In this regard, our work sets a good example for microarray-based discovery of prognostic gene sets. We included more than 1,700 samples in the analysis and applied complete external validation to avoid data over-fitting. Thus, we believe that gene sets found in our analysis are truly prognostic in breast cancer and not just a noisy discovery. Finally, although we focused only on breast cancer datasets in this work, our gene sets approach is equally applicable to other types of cancer or to studies that develop molecular signatures for predicting drug sensitivity of each patient to cancer drugs. We expect that, like gene set enrichment analysis and similar tools that have become useful for gene expression data analysis [21,22], a gene sets approach will be useful for developing prognostic signatures for outcome prediction [23].

## Conclusion

The gene sets approach is an effective tool for selecting a prognostic gene set as well as for understanding the underlying biology for different patients' outcomes. By applying a bottom-up approach with many gene sets, we could identify the biological processes and pathways that are important for prognosis of breast cancer patients. The importance of cell proliferation signatures in breast cancer prognosis has been repeatedly discovered, but our approach reinforces these previous findings [1,2,13,15,16,18,24,30,38]. Additionally, our approach is applicable to other types of cancer in which prognostic gene sets are less developed than breast cancer.

## Methods

### Datasets

We downloaded breast cancer gene expression datasets with clinical information from the gene expression omnibus [39], Stanford microarray database [40], or author's individual web pages [1,2,15-20,26,27,38,41]. See Table 1 for a complete list of datasets and their sources. We analyzed 12 datasets comprising 1,756 tissue samples.

### Gene sets

We prepared gene sets from diverse sources including gene ontology (GO) terms [42], GenMAPP [43] and KEGG pathways [44], and InterPro protein domain information [45] using the Affymetrix annotation file (2006 November version) downloaded from the Affymetrix web site (Table 2)[46]. We limited the gene set size between five and two thousands. We also included 32 well-known prognostic gene sets for breast and other cancers (Table 3). For those 32 gene sets, we created a nomenclature for each gene set by combining the PubMed id of the reference and the source of the gene set in the reference. For example, 11823860_ST2 represents a gene set from the Supplementary Table 2 from van't Veer et al. [13] with a PubMed id of 118323860. The number of gene sets in each category is shown in Table 2.

### Preprocessing of microarray data

The datasets that we analyzed included both single-channel Affymetrix and dual-channel cDNA microarray platforms. We used a gene symbol as a common identifier to map probe IDs across different platforms. When we mapped a gene set between two arrays, we used only genes common to both arrays. To analyze Affymetrix datasets, we consistently used expression values computed by MAS5 algorithms to ensure similar processing, normalized each sample by a global mean method to a target density of 1,000, floored low expression values to 100, log-transformed each value by base two, merged replicate probes for the same gene by an average value, and finally mean-centered each gene within a dataset [47]. To analyze cDNA datasets, we initially filtered out missing values when the percentage of missing values was greater than 30%, imputed missing values by the k-nearest neighbor method, merged replicate probes by an average value, and finally mean-centered each gene. We used the GEPAS web service [48] to filter and impute missing values [49].

### Statistical analysis

For each dataset and gene set, we applied k-means clustering with k = 2, 3, 4, 5, and 6 to divide each sample into two, three, four, five, or six groups based on the gene expression pattern of the gene set and applied the log-rank test to infer the statistical significance of differences in survival between the groups. We used a Kaplan-Meier plot to show the differences in survival. We applied the nearest-centroid prediction rule, one of the simplest class prediction methods, to estimate the accuracy of prediction for each patient's outcome [7]. To briefly describe, the nearest-centroid prediction rule first calculates a centroid for each group. The centroid is the average gene expression for each gene in each group. Then, with a new sample, the method calculates two distances between the gene expression value of the new sample and each of the two centroids and assigns the new sample to the group with the smaller distance. For each gene set, we defined two average profiles (good and poor) as vectors of the average expression values of genes in a gene set in patients with good and poor prognoses. Good prognosis patients were defined as relapse-free or overall survival over five years, whereas poor prognosis patients were deceased within five years. We classified each patient in the validation set according to the Euclidean distance between the gene expression of the patient and the two average profiles. We performed external validation using six large datasets containing more than 100 samples. For external validation, we calculated two average (good and poor) profiles using only samples in one dataset and predicted patient outcomes in the other five datasets and performed external validation for all training-testing pairs of six datasets (30 pairs). We used R language [50] for statistical analysis and python programming language [51] for data processing.

## List of abbreviations used

GEO: Gene expression omnibus; GSE: Gene expression Series; GO: Gene ontology; BP: Biological processes; MF: Molecular functions; PW: pathway, BR: Breast cancer prognostic signature; ER: estrogen receptor.

## Author's contributions

SYK designed the study, collected datasets, performed bioinformatics analyses, and drafted the manuscript. YSK designed the study and wrote the manuscript. Both authors read and approved the final manuscript.

## Supplementary Material

Additional file 1 **Additional data file 1 **contains Supplementary tables (1–4) showing top 20 prognostic gene sets from three, four, five, and six means clustering of the 12 data sets. Click here for file

Additional file 2 **Additional data file 2 **contains tables (5–7) showing adjusted hazard ratios of the gene set 11823860_ST2 for available clinical parameters in Sotiriou_2, van de Vijver, and Sorlie_1 datasets, respectively. Click here for file

Additional file 3 **Additional data file 3 **is a Supplementary table (8) showing top 20 gene sets with high prediction accuracy in independent validation using nine datasets. Click here for file

## References

[B1] van de Vijver MJ, He YD, van't Veer LJ, Dai H, Hart AA, Voskuil DW, Schreiber GJ, Peterse JL, Roberts C, Marton MJ (2002). A gene-expression signature as a predictor of survival in breast cancer.. The New England journal of medicine.

[B2] Wang Y, Klijn JG, Zhang Y, Sieuwerts AM, Look MP, Yang F, Talantov D, Timmermans M, Meijer-van Gelder ME, Yu J (2005). Gene-expression profiles to predict distant metastasis of lymph-node-negative primary breast cancer.. Lancet.

[B3] Chang HY, Sneddon JB, Alizadeh AA, Sood R, West RB, Montgomery K, Chi JT, van de Rijn M, Botstein D, Brown PO (2004). Gene expression signature of fibroblast serum response predicts human cancer progression: similarities between tumors and wounds.. PLoS biology.

[B4] Alizadeh AA, Eisen MB, Davis RE, Ma C, Lossos IS, Rosenwald A, Boldrick JC, Sabet H, Tran T, Yu X (2000). Distinct types of diffuse large B-cell lymphoma identified by gene expression profiling.. Nature.

[B5] Buyse M, Loi S, van't Veer L, Viale G, Delorenzi M, Glas AM, d'Assignies MS, Bergh J, Lidereau R, Ellis P (2006). Validation and clinical utility of a 70-gene prognostic signature for women with node-negative breast cancer.. Journal of the National Cancer Institute.

[B6] Paik S, Shak S, Tang G, Kim C, Baker J, Cronin M, Baehner FL, Walker MG, Watson D, Park T (2004). A multigene assay to predict recurrence of tamoxifen-treated, node-negative breast cancer.. The New England journal of medicine.

[B7] Michiels S, Koscielny S, Hill C (2005). Prediction of cancer outcome with microarrays: a multiple random validation strategy.. Lancet.

[B8] Eden P, Ritz C, Rose C, Ferno M, Peterson C (2004). "Good Old" clinical markers have similar power in breast cancer prognosis as microarray gene expression profilers.. Eur J Cancer.

[B9] Ransohoff DF (2005). Bias as a threat to the validity of cancer molecular-marker research.. Nature reviews.

[B10] Ein-Dor L, Kela I, Getz G, Givol D, Domany E (2005). Outcome signature genes in breast cancer: is there a unique set?. Bioinformatics (Oxford, England).

[B11] Ein-Dor L, Zuk O, Domany E (2006). Thousands of samples are needed to generate a robust gene list for predicting outcome in cancer.. Proceedings of the National Academy of Sciences of the United States of America.

[B12] Chang HY, Nuyten DS, Sneddon JB, Hastie T, Tibshirani R, Sorlie T, Dai H, He YD, van't Veer LJ, Bartelink H (2005). Robustness, scalability, and integration of a wound-response gene expression signature in predicting breast cancer survival.. Proceedings of the National Academy of Sciences of the United States of America.

[B13] van 't Veer LJ, Dai H, van de Vijver MJ, He YD, Hart AA, Mao M, Peterse HL, van der Kooy K, Marton MJ, Witteveen AT (2002). Gene expression profiling predicts clinical outcome of breast cancer.. Nature.

[B14] Naderi A, Teschendorff AE, Barbosa-Morais NL, Pinder SE, Green AR, Powe DG, Robertson JF, Aparicio S, Ellis IO, Brenton JD (2007). A gene-expression signature to predict survival in breast cancer across independent data sets.. Oncogene.

[B15] Sotiriou C, Wirapati P, Loi S, Harris A, Fox S, Smeds J, Nordgren H, Farmer P, Praz V, Haibe-Kains B (2006). Gene expression profiling in breast cancer: understanding the molecular basis of histologic grade to improve prognosis.. Journal of the National Cancer Institute.

[B16] Pawitan Y, Bjohle J, Amler L, Borg AL, Egyhazi S, Hall P, Han X, Holmberg L, Huang F, Klaar S (2005). Gene expression profiling spares early breast cancer patients from adjuvant therapy: derived and validated in two population-based cohorts.. Breast Cancer Res.

[B17] West M, Blanchette C, Dressman H, Huang E, Ishida S, Spang R, Zuzan H, Olson JA, Marks JR, Nevins JR (2001). Predicting the clinical status of human breast cancer by using gene expression profiles.. Proceedings of the National Academy of Sciences of the United States of America.

[B18] Sotiriou C, Neo SY, McShane LM, Korn EL, Long PM, Jazaeri A, Martiat P, Fox SB, Harris AL, Liu ET (2003). Breast cancer classification and prognosis based on gene expression profiles from a population-based study.. Proceedings of the National Academy of Sciences of the United States of America.

[B19] Miller LD, Smeds J, George J, Vega VB, Vergara L, Ploner A, Pawitan Y, Hall P, Klaar S, Liu ET (2005). An expression signature for p53 status in human breast cancer predicts mutation status, transcriptional effects, and patient survival.. Proceedings of the National Academy of Sciences of the United States of America.

[B20] Bild AH, Yao G, Chang JT, Wang Q, Potti A, Chasse D, Joshi MB, Harpole D, Lancaster JM, Berchuck A (2006). Oncogenic pathway signatures in human cancers as a guide to targeted therapies.. Nature.

[B21] Subramanian A, Tamayo P, Mootha VK, Mukherjee S, Ebert BL, Gillette MA, Paulovich A, Pomeroy SL, Golub TR, Lander ES (2005). Gene set enrichment analysis: a knowledge-based approach for interpreting genome-wide expression profiles.. Proceedings of the National Academy of Sciences of the United States of America.

[B22] Kim SY, Volsky DJ (2005). PAGE: parametric analysis of gene set enrichment.. BMC bioinformatics.

[B23] Goeman JJ, Oosting J, Cleton-Jansen AM, Anninga JK, van Houwelingen HC (2005). Testing association of a pathway with survival using gene expression data.. Bioinformatics (Oxford, England).

[B24] Teschendorff AE, Naderi A, Barbosa-Morais NL, Pinder SE, Ellis IO, Aparicio S, Brenton JD, Caldas C (2006). A consensus prognostic gene expression classifier for ER positive breast cancer.. Genome biology.

[B25] Perou CM, Sorlie T, Eisen MB, van de Rijn M, Jeffrey SS, Rees CA, Pollack JR, Ross DT, Johnsen H, Akslen LA (2000). Molecular portraits of human breast tumours.. Nature.

[B26] Sorlie T, Perou CM, Tibshirani R, Aas T, Geisler S, Johnsen H, Hastie T, Eisen MB, van de Rijn M, Jeffrey SS (2001). Gene expression patterns of breast carcinomas distinguish tumor subclasses with clinical implications.. Proceedings of the National Academy of Sciences of the United States of America.

[B27] Sorlie T, Tibshirani R, Parker J, Hastie T, Marron JS, Nobel A, Deng S, Johnsen H, Pesich R, Geisler S (2003). Repeated observation of breast tumor subtypes in independent gene expression data sets.. Proceedings of the National Academy of Sciences of the United States of America.

[B28] Dudoit S, Fridlyand J, Speed TP (2002). Comparison of discrimination methods for the classification of tumors using gene expression data.. Journal of the American Statistical Association.

[B29] Whitfield ML, George LK, Grant GD, Perou CM (2006). Common markers of proliferation.. Nature Reviews Cancer.

[B30] Fan C, Oh DS, Wessels L, Weigelt B, Nuyten DS, Nobel AB, van't Veer LJ, Perou CM (2006). Concordance among gene-expression-based predictors for breast cancer.. The New England journal of medicine.

[B31] Chang JT, Nevins JR (2006). GATHER: a systems approach to interpreting genomic signatures.. Bioinformatics (Oxford, England).

[B32] Simon R (2006). Development and evaluation of therapeutically relevant predictive classifiers using gene expression profiling.. Journal of the National Cancer Institute.

[B33] Pepe MS, Janes H, Longton G, Leisenring W, Newcomb P (2004). Limitations of the odds ratio in gauging the performance of a diagnostic, prognostic, or screening marker.. American journal of epidemiology.

[B34] Ioannidis JP (2007). Is molecular profiling ready for use in clinical decision making?. The oncologist.

[B35] Pepe MS (2005). Evaluating technologies for classification and prediction in medicine.. Statistics in medicine.

[B36] Ioannidis JP (2005). Microarrays and molecular research: noise discovery?. Lancet.

[B37] Reis-Filho JS, Westbury C, Pierga JY (2006). The impact of expression profiling on prognostic and predictive testing in breast cancer.. J Clin Pathol.

[B38] Weigelt B, Hu Z, He X, Livasy C, Carey LA, Ewend MG, Glas AM, Perou CM, Van't Veer LJ (2005). Molecular portraits and 70-gene prognosis signature are preserved throughout the metastatic process of breast cancer.. Cancer research.

[B39] Gene expression omnibus (GEO). http://www.ncbi.nlm.nih.gov/GEO/.

[B40] Stanford Microarray Database. http://genome-www5.stanford.edu/.

[B41] Oh DS, Troester MA, Usary J, Hu Z, He X, Fan C, Wu J, Carey LA, Perou CM (2006). Estrogen-regulated genes predict survival in hormone receptor-positive breast cancers.. J Clin Oncol.

[B42] The Gene Ontology. http://www.geneontology.org.

[B43] GenMAPP. http://www.genmapp.org.

[B44] KEGG: Kyoto Encyclopedia of Genes and Genomes. http://www.genome.up/kegg/.

[B45] InterPro. http://www.ebi.ac.uk/interpro/.

[B46] Affymetrix..

[B47] Kim SY, Kim Y (2006). Genome-wide prediction of transcriptional regulatory elements of human promoters using gene expression and promoter analysis data.. BMC bioinformatics.

[B48] GEPAS: Gene Expression Pattern Analysis Suite. http://gepas.bioinfo.cipf.es/.

[B49] Vaquerizas JM, Conde L, Yankilevich P, Cabezon A, Minguez P, Diaz-Uriarte R, Al-Shahrour F, Herrero J, Dopazo J (2005). GEPAS, an experiment-oriented pipeline for the analysis of microarray gene expression data.. Nucleic acids research.

[B50] The R Project for Statistical Computing. http://www.r-project.org/.

[B51] Python Programming Language. http://www.python.org.

[B52] Huang E, Cheng SH, Dressman H, Pittman J, Tsou MH, Horng CF, Bild A, Iversen ES, Liao M, Chen CM (2003). Gene expression predictors of breast cancer outcomes.. Lancet.

[B53] Zhao H, Langerod A, Ji Y, Nowels KW, Nesland JM, Tibshirani R, Bukholm IK, Karesen R, Botstein D, Borresen-Dale AL (2004). Different gene expression patterns in invasive lobular and ductal carcinomas of the breast.. Molecular biology of the cell.

[B54] Glinsky GV, Higashiyama T, Glinskii AB (2004). Classification of human breast cancer using gene expression profiling as a component of the survival predictor algorithm.. Clin Cancer Res.

[B55] Glinsky GV, Berezovska O, Glinskii AB (2005). Microarray analysis identifies a death-from-cancer signature predicting therapy failure in patients with multiple types of cancer.. The Journal of clinical investigation.

[B56] Schuetz CS, Bonin M, Clare SE, Nieselt K, Sotlar K, Walter M, Fehm T, Solomayer E, Riess O, Wallwiener D (2006). Progression-specific genes identified by expression profiling of matched ductal carcinomas in situ and invasive breast tumors, combining laser capture microdissection and oligonucleotide microarray analysis.. Cancer research.

