# Supplementary material for: A gene sets approach for identifying prognostic gene signatures for outcome prediction
Source: BMC Genomics. 2008 Apr 16;9:177. doi: 10.1186/1471-2164-9-177 (PMC2364634; doi:10.1186/1471-2164-9-177)
Supplement: Additional file 1 — Additional data file 1 contains Supplementary tables (1–4) showing top 20 prognostic gene sets from three, four, five, and six means clustering of the 12 data sets. [file 1471-2164-9-177-S1.rtf]

SupplementaryTable 1. Top 20 prognostic gene sets identified by three-means clustering in breast cancer gene expression datasets
Gene set	*category	Bild	Miller	Oh	Pawitan	Sorlie_1	Sorlie_2	Sotirou_1	Sotiriou_2	van de Vijver	Wang	Weigelt	West	#frequency	%mean	
12917485_ST9	BR	3.18	15.51	7.87	16.86	17.94	4.17	10.7	13.34	41.6	8.49	0.7	1.06	8	11.79	
cell cycle 	BP	6.55	10.08	10.51	15.5	10.82	0.18	3.62	11.38	47.82	8.4	1.52	0.01	8	10.53	
mitosis 	BP	7.31	11.07	7.56	14.9	10.68	1.1	2.4	12.07	36.12	13.51	1.61	0.01	8	9.86	
protein domain specific binding	MF	7.38	16.34	0.02	7.49	0.44	4.65	12.24	10.71	13.86	6.18	12.43	1.1	8	7.74	
11823860_ST2	BR	3.46	8.45	14.3	16.43	14.32	0.73	5.63	21.5	54.99	17.41	2.21	2.52	7	13.5	
Cell_cycle_KEGG_GenMAPP	PW	8.96	12.04	3.51	14.85	9.7	0.49	3.92	16	46.45	10.35	2.06	1.01	7	10.78	
12917485_ST8	BR	0.93	12.06	7.24	25.11	16.05	3.26	2.33	11.27	37.21	9.13	1.64	1.38	7	10.63	
cAMP biosynthesis 	BP	0.27	11.39	22.03	5.95	11.22	17.31	9.21	0.48	38.19	6.63	1.46	1.19	7	10.44	
16478745_ST1	BR	3.4	14.52	6.72	17.45	12.34	1.57	4.66	15.14	35.77	8.99	1.27	0.55	7	10.2	
15591335	BR	0.5	9.05	9.02	15.45	10.38	7	0.94	16.98	38.18	3.1	3.37	3.5	7	9.79	
cell division 	BP	6.72	15.14	5.23	15.72	8.09	1.12	2.34	14.49	35.56	10.54	0.6	0.93	7	9.71	
electron transport 	BP	1.13	11.59	10.19	7.55	1.52	1.07	3.4	7.13	42.77	10.46	6.01	2.98	7	8.82	
16280042_AF1	BR	6.16	16.28	1.12	16.79	6.8	0.12	1.72	11.1	30.63	6.3	0.23	1.45	7	8.22	
14-3-3 protein	IP	6.04	17.32	4.01	7.52	5.02	3.02	12.71	10.25	14.92	6.27	5.74	1.6	7	7.87	
Circadian_Exercise_GenMAPP	PW	1.32	12.66	8.56	8.4	9.89	2.08	8.6	0.26	23.41	9.88	2.74	4.06	7	7.66	
Forkhead-associated (FHA)	IP	7.25	13.07	4.96	12.57	6.07	3	0.4	6.18	23.94	8.77	0.34	0.97	7	7.29	
Valine, leucine and isoleucine degradation_KEGG	PW	4.3	12.26	3.69	9.13	7.95	10.98	7.07	3.47	17.06	2.96	1.32	7.18	7	7.28	
Ubiquitin-conjugating enzymes	IP	9.54	11.65	9.48	16.67	2.19	0.42	2.24	11.48	11.21	6.79	3.4	1.31	7	7.2	
organic anion transporter activity	MF	6.9	13.57	8.03	7.04	2.95	8.24	0.35	10.71	5.36	18.19	0.82	1.64	7	6.98	
Major intrinsic protein	IP	0.38	10.21	10.06	11.53	0.96	6.13	16.16	0.22	6.47	10.34	1.6	3.2	7	6.44	
Values are chi-square values from log-rank test.
#frequency: The number of cases in which chi-square value is over 5.99
*category: BP-GO Biological Processes, BR-Breast cancer prognostic signatures, MF-GO Molecular Function, PW-KEGG and GenMAPP pathways, IP-InterPro domains
%mean: Mean of 12 chi-square values


Supplementary Table 2. Top 20 prognostic gene sets identified by four-means clustering in breast cancer gene expression datasets
Gene set	category	Bild	Miller	Oh	Pawitan	Sorlie_1	Sorlie_2	Sotiriou_1	Sotirou_2	Van de Vijver	Wang	Weigelt	West	#frequency	mean	
11823860_ST2	br	7.35	13.16	18.82	16.5	18.71	2.41	6.28	17.71	55.71	23.76	8.57	2.5	8	15.96	
12917485_ST9	br	3.11	21.83	11.55	16.65	17.27	3.05	12.27	18.17	45.07	8.1	3.38	1.06	8	13.46	
16141321_SDC2	br	2.78	19.87	9.6	19.48	12.26	4.4	8.12	12.93	47.68	20.84	2.01	1.2	8	13.43	
G1_to_S_cell_cycle_Reactome_GenMAPP	pw	8.36	13.3	10.9	18.52	8.01	0.86	6.08	15.26	50.64	13.94	1.21	2.34	8	12.45	
phosphoinositide-mediated signaling	bp	5.54	11.83	6.27	13.04	2.22	9.57	9.65	12.24	38.47	13.32	12.09	5.9	8	11.68	
Ubiquitin-conjugating enzymes	ip	8.24	12.63	11.47	17.06	13.72	2.49	1.69	14.9	13.4	14.17	4.41	1.34	8	9.63	
exonuclease activity	mf	1.12	10.21	6.77	11.65	0.32	7.98	9.03	9.43	27.79	14.86	3.18	9.72	8	9.34	
Arachidonic acid metabolism_KEGG	pw	15.16	14.42	9.96	8.24	5.94	1.76	10.22	9.3	14.52	9.19	3.81	2.8	8	8.78	
ligand-dependent nuclear receptor transcription coactivator activity	mf	4.19	9.12	9.98	12.58	9.1	12.42	3.56	3.47	11.05	5.11	12.76	9.02	8	8.53	
16478745_ST1	br	5.91	17.31	20.66	15.38	12.68	2.01	5.77	27.61	43.89	23.85	2.24	2.67	7	15	
Cell_cycle_KEGG_GenMAPP	pw	9.96	7.59	12.47	15.37	11.14	1.24	6.46	24.67	53.67	13.46	2.82	6.31	7	13.76	
caspase inhibitor activity	mf	1.82	8.76	10.51	14.11	1.7	4.06	2.22	19.13	43.61	34.32	8.71	3.32	7	12.69	
12917485_ST7	br	4.78	16.33	8.86	13.6	10.89	7.24	6.35	24.26	30.72	12.8	7.2	2.72	7	12.15	
12490681_70	br	3.28	24.01	10.7	15.35	9.28	1.24	6.28	9.03	46.11	9.14	5.22	3.89	7	11.96	
15591335	br	2.01	11.77	8.33	13.59	11.32	7.43	2.99	12.46	44.32	21.11	3.47	1.38	7	11.68	
Kinesin, motor region	ip	1.01	14.1	10.2	15.06	5.04	4.07	2.57	20.22	43.6	9.99	0.35	13.01	7	11.6	
nucleobase, nucleoside, nucleotide and nucleic acid metabolism	bp	2.35	8.34	8.74	11.29	6.66	40.04	0.07	9.53	37.65	10	0.63	1.19	7	11.37	
DNA replication	bp	7.03	11.57	5.95	16.1	10.15	3.06	12.88	13.78	36.09	9.12	7.34	1.92	7	11.25	
mitotic spindle checkpoint	bp	4.44	12.75	7.66	8.12	10.81	0.9	4.86	12.78	42.6	8.86	14.02	4.82	7	11.05	
magnesium ion binding	mf	0.63	11.88	14.63	18.37	17.15	0.92	14.3	4.52	35.37	8.64	0.58	4.17	7	10.93	
#frequency: The number of cases in which chi-square value is over 7.815


Supplementary Table 3. Top 20 prognostic gene sets identified by five-means clustering in breast cancer gene expression datasets
geneset	*category	Bild	Miller	Oh	Pawitan	Sorlie_1	Sorlie_2	Sotiriou_1	Sotiriou_2	van de Vijver	Wang	West	Wiegelt	#frequency	%mean	
12917485_ST7	BR	3.94	35.68	10.95	17.91	10.45	9.42	3.68	22.57	44.15	17.29	3.49	16.97	8	16.37	
16141321_SDC2	BR	6.4	18.96	14.86	22	14.21	10.79	4.95	15.77	50.6	22.2	0.84	3.78	8	15.45	
glycolysis	BP	7.03	9.94	13.2	13.25	9.23	39.73	16.24	0.72	35.79	10.65	2.31	10.01	8	14.01	
16707453_ST3	BR	2.4	35.85	14.58	11.16	19.99	10.97	10.17	10.77	38.34	7.92	1.04	4.1	8	13.94	
magnesium ion binding	MF	1.78	15.47	15.01	16.25	23.16	3.37	18.73	10.32	43.74	12.75	2.18	1.84	8	13.71	
calcium ion homeostasis	BP	2.23	19.77	3.22	12.79	12.4	3.61	10	15.5	22.72	12.78	1.43	35.42	8	12.65	
Butanoate metabolism_KEGG	PW	4.62	12.99	15.47	12.1	10.28	15.01	3.22	7.12	37.29	12.45	13.48	5.14	8	12.43	
Cell_cycle_KEGG_GenMAPP	PW	15.54	18.38	4.34	24.1	17.97	1.2	8.47	22.65	48.32	18	6.92	2.18	7	15.67	
12917485_ST9	BR	7.8	28.71	13.27	20.99	10.53	6.07	13.32	22.59	43.04	9.21	5.05	5.97	7	15.55	
11823860_ST2	BR	9.32	12.6	21.37	18.59	18.07	2.16	7.68	24.14	48.26	19.01	2	2.49	7	15.47	
12490681_70	BR	7.94	18.24	9.42	18.25	13.14	2.92	6.73	26.92	47.18	9.73	5.96	15.87	7	15.19	
11823860_ST3	BR	2.94	28.66	11.46	17.54	20.58	2.15	8.12	13.77	37.22	15.41	3.72	2.9	7	13.71	
transporter activity	MF	4.86	10.71	13.48	29.03	16.5	3.53	13.34	1.28	48.07	5.2	4.53	9.83	7	13.36	
microtubule-based movement	BP	4.82	14.13	10.68	19.22	14.35	6.89	3.19	17.8	40.77	13.22	8.55	2.31	7	13	
ATP binding	MF	4.83	13.81	10.32	27.59	10.58	3.22	6.43	19.11	41.28	11.99	2.57	3.41	7	12.93	
14737219_USR	BR	4.35	23.94	10.54	14.77	15.6	3.85	2.37	19.79	40.45	11.4	3.85	3.72	7	12.89	
cAMP biosynthesis	BP	0.85	12.87	23.73	18.59	8.47	18.26	7.17	8.42	30.36	10.89	10.41	3.16	7	12.76	
Nuclear_Receptors_GenMAPP	PW	10.92	17.28	12.56	16.56	18.15	10.6	5.42	1.63	44.23	7.34	3.13	2.15	7	12.5	
chromatin binding	MF	3.4	9.64	17.42	17.1	13.87	5.21	2.76	6.35	42.66	9.82	12.39	8.06	7	12.39	
phosphoinositide-mediated signaling	BP	5.8	14.53	6.73	14.05	2.74	8.78	11.26	12.98	43.79	11.77	3.08	12.4	7	12.32	
#frequency: The number of cases in which chi-square value is over 9.49


Supplementary Table 4. Top 20 prognostic gene sets identified by six-means clustering in breast cancer gene expression datasets
Gene set	category	Bild	Miller	Oh	Pawitan	Sorlie_1	Sorlie_2	Sotiriou_1	Sotiriou_2	van de Vijver	Wang	Weigelt	West	#frequency	mean	
Cell_cycle_KEGG_GenMAPP	pw	18.38	17.93	11.75	20.74	14.93	1.63	5.27	28.73	51.34	18.77	4.2	5.16	8	16.57	
transcription factor activity	mf	2.68	26.02	18.64	12.24	30.59	6.99	17.74	17.67	36.26	16.11	9.56	3.33	8	16.49	
kinase activity	mf	2.53	34.42	11.38	25.58	14.75	2.93	11.19	14.61	40.67	15.64	4.07	4.15	8	15.16	
16273092_SRC	br	1.74	39.01	11.5	21.94	14.25	8.6	11.1	18.73	26.25	20.1	2.82	4.89	8	15.08	
12917485_ST9	br	3.84	18.77	12.75	21.13	14.57	4.79	14.46	22.41	44.97	11.14	7.01	5.05	8	15.08	
microtubule motor activity	mf	5.38	14.21	12.25	20.88	13.45	15.52	3.49	26.7	22.45	20.16	2.34	9.36	8	13.85	
protein homodimerization activity	mf	3.14	15.33	14.98	12.55	12.69	4.3	2.51	2.86	28.54	25.84	17.16	14.52	8	12.87	
protein domain specific binding	mf	11.27	13.72	6.28	16.47	8.02	11.36	20.49	13.31	17.13	6.06	12.63	6.47	8	11.93	
Janus kinase activity	mf	13.32	12.17	12.26	18.11	4.08	20.63	14.29	5.19	14.37	12.48	6.81	2.37	8	11.34	
11823860_ST2	br	4.79	17.12	15.03	23.41	20.49	3.58	6.23	26.94	55.3	30	2.8	3.31	7	17.42	
16141321_SDC2	br	8.42	31.7	8.56	23.02	14.27	4.9	8.04	22.27	53.47	12.28	12.7	0.44	7	16.67	
magnesium ion binding	mf	0.95	27.88	25.78	23.84	12.41	4.95	24.62	13.09	45.87	8.6	2.4	5.51	7	16.33	
12917485_ST7	br	8.14	32.23	16.37	21.93	11.69	7.06	5.21	24.29	46.05	16.21	3.68	2.81	7	16.3	
G1_to_S_cell_cycle_Reactome_GenMAPP	pw	13.79	17.1	8.83	15.2	17.06	0.62	6.48	23.91	54.6	11.27	6.77	1.16	7	14.73	
mitotic spindle checkpoint	bp	4.36	17.72	14.75	11.34	9.65	9.16	7.02	23.57	40.65	17.33	13.92	6.29	7	14.65	
brain development	bp	3.95	13.57	11.46	27.59	17.09	17.64	5.07	2.88	45.88	15.34	5.35	6.73	7	14.38	
Butanoate metabolism_KEGG	pw	12.89	15.05	14.67	10.56	29.98	14.98	3.8	11.97	38.42	9.41	4.29	1.87	7	13.99	
double-stranded DNA binding	mf	1.41	28.35	13.87	14.51	15.65	13.78	6.27	13.27	42.6	7.24	5.72	4.73	7	13.95	
transcription	bp	3.29	26	12.69	19.62	13.22	12.54	4.42	19.81	35.46	10.5	5.62	4.22	7	13.95	
Nuclear_Receptors_GenMAPP	pw	1.71	27.24	13.38	11.38	20.53	14.27	4.54	3.3	47.38	13.56	6.13	3.25	7	13.89	
#frequency: The number of cases in which chi-square value is over 11.07
