# Supplementary material for: A gene sets approach for identifying prognostic gene signatures for outcome prediction
Source: BMC Genomics. 2008 Apr 16;9:177. doi: 10.1186/1471-2164-9-177 (PMC2364634; doi:10.1186/1471-2164-9-177)
Supplement: Additional file 2 — Additional data file 2 contains tables (5–7) showing adjusted hazard ratios of the gene set 11823860_ST2 for available clinical parameters in Sotiriou_2, van de Vijver, and Sorlie_1 datasets, respectively. [file 1471-2164-9-177-S2.rtf]

Supplementary Table 5. Hazard ratios (with 95% confidence interval) and P values for individual risk factors and risk classifications with Sotiriou_2 data set and 11823860_ST2 gene set
Risk factor or classification	Hazard ratios and P values	
Grade	1.28 (0.938-1.75), p = 0.119	
Lymph Node	0.96 (0.457-2.03), p = 0.92	
Tumor size	1.53 (1.25-1.87), p = 3.2e-5	
Age	1.00 (0.98-1.03), p = 0.76	
ER status	1.42 (0.407-1.21), p = 0.204	
Gene signature	5.13 (1.6-16.4), p = 0.00592	
		
Grade + gene signature	6.45 (1.429-29.10), p = 0.015	
Lymph node + gene signature	5.47 (1.679-17.83), p = 0.0048	
Tumor size + gene signature	4.54 (1.39-14.86), p = 1.2e-02	
Age + gene signature	5.28 (1.64-16.99), p = 0.0053	
ER status + gene signature	7.10 (1.903-26.46), p = 0.0035	


Supplementary Table 6. Hazard ratios (with 95% confidence interval) and P values for individual risk factors and risk classifications with van de Vijer data set and 11823860_ST2 gene set
Risk factor or classification	Hazard ratios and P values	
Lymph node	1.14 (0.563-1.37), p = 0.56	
ER status	0.304 (0.195-0.476), p = 1.88e-7	
NIH criteria	0.328 (0.0456-2.36), p = 0.268	
St. Gallen criteria	0.142 (0.0198-1.02), p = 0.0526	
Gene signature	62.3 (17.7-219), p = 1.12e-10	
		
Lymph node + gene signature	51.18 (14.10-185.76), p = 2.2e-9	
ER status + gene signature	30.52 (7.79-119.66), p = 9.4e-7	
NIH criteria + gene signature	59.7 (16.89-211.04), p = 2.2e-10	
St. Gallen criteria + gene signature	51.18 (14.10-185.76), p = 2.2e-9	


Supplementary Table 7. Hazard ratios (with 95% confidence interval) and P values for individual risk factors and risk classifications with Sorlie_1 data set and 11823860_ST2 gene set
Risk factor or classification	Hazard ratios and P values	
Grade	2.03 (1.13-3.65), p = 0.0179	
Lymph node	1.09 (0.914-1.75), p = 0.71	
Tumor size	1.72 (0.98-3.03), p = 0.0581	
Age	1.01 (0.98-1.03), p = 0.505	
ER status	0.216 (0.097-0.479), p = 1.7e-4	
Gene signature	6.84 (1.75-26.7), p = 0.00568	
		
Grade + gene signature	4.22 (0.99-18.00), p = 0.052	
Lymph node + gene signature	6.82 (1.74-26.73), p = 0.00592	
Tumor size + gene signature	5.69 (1.43-22.62), p = 0.0013 	
Age + gene signature	6.79 (1.73-26.66), p = 0.0061	
ER status + gene signature	5.05 (1.30-19.52), p = 0.019	
