# Supplementary material for: A gene sets approach for identifying prognostic gene signatures for outcome prediction
Source: BMC Genomics. 2008 Apr 16;9:177. doi: 10.1186/1471-2164-9-177 (PMC2364634; doi:10.1186/1471-2164-9-177)
Supplement: Additional file 3 — Additional data file 3 is a Supplementary table (8) showing top 20 gene sets with high prediction accuracy in independent validation using nine datasets. [file 1471-2164-9-177-S3.rtf]

Supplementary Table 8. Top 20 gene sets with high prediction accuracy calculated using nine datasets
Gene set	category	GTG	GTP	PTG	PTP	accuracy	sensitivity	specificity	
11823860_ST2 	br	5472	2464	1191	1553	0.6578	0.6895	0.566	
12917485_ST9 	br	5435	2501	1208	1536	0.6527	0.6849	0.5598	
Kinase-associated, C-terminal 	ip	1230	591	231	306	0.6514	0.6755	0.5698	
Glutamine amidotransferase class-I 	ip	2292	1064	429	479	0.6499	0.683	0.5275	
Glutamine amidotransferase, class-II 	ip	2277	1079	414	494	0.6499	0.6785	0.5441	
spindle organization and biogenesis 	bp	2939	1561	475	830	0.6493	0.6531	0.636	
Cation efflux protein 	ip	1784	888	349	503	0.649	0.6677	0.5904	
negative regulation of translational initiation 	bp	1231	590	240	297	0.648	0.676	0.5531	
eukaryotic initiation factor 4E binding 	mf	1231	590	240	297	0.648	0.676	0.5531	
Cell_cycle_KEGG_GenMAPP 	pw	5300	2636	1149	1595	0.6456	0.6678	0.5813	
mitotic checkpoint 	bp	3654	1842	730	1016	0.6448	0.6648	0.5819	
cell division 	bp	5254	2682	1129	1615	0.6432	0.662	0.5886	
ligase activity 	mf	5425	2511	1301	1443	0.6431	0.6836	0.5259	
folic acid and derivative biosynthesis 	bp	1218	603	240	297	0.6425	0.6689	0.5531	
Ndr family 	ip	1257	564	282	255	0.6412	0.6903	0.4749	
17076897_ADF3 	br	5189	2747	1086	1658	0.6411	0.6539	0.6042	
transferase activity 	mf	5414	2522	1315	1429	0.6407	0.6822	0.5208	
11823860_ST3 	br	5434	2502	1337	1407	0.6405	0.6847	0.5128	
regulation of bone mineralization 	bp	1246	575	274	263	0.6399	0.6842	0.4898	
Cyclin, C-terminal 	ip	5250	2686	1161	1583	0.6398	0.6615	0.5769	
*category: br – breast cancer gene set; mf – molecular functions; bp – biological processes
**GTG - Good prognosis group predicted as Good; GTP – Good prognosis group predicted as Poor; PTG – Poor prognosis group identified as Good; PTP – Poor prognosis group identified as Poor
^accuracy = (GTG+ PTP)/(GTP+GTP+PTG+PTP); sensitivity = GTG/(GTG+GTP); specificity = PTP/(PTG+PTP)
